# Supplementary material for: Understanding the diagnostic delays and pathways for diabetes in eastern Uganda: A qualitative study
Source: PLoS One. 2021 Apr 21;16(4):e0250421. doi: 10.1371/journal.pone.0250421 (PMC8059813; doi:10.1371/journal.pone.0250421)
Supplement: S1 Text — (DOCX) [file pone.0250421.s001.docx]

**S1 Text. Preliminary Template**

1. **Appraisal interval**
   1. Short vs long appraisal
   2. Symptom experience
      1. Minor discomfort
      2. Escalation of symptoms
      3. Very distressing symptoms
      4. Symptom attribution
   3. Self-care
      1. Resting it out
      2. Rehydration and showers
      3. Herbal medication
   4. Lay consultation
      1. Attribution of symptoms
      2. Self-management support
      3. Advice to seek professional help
2. **Diagnostic interval**
   1. Short vs long diagnostic interval
   2. Type of health provider
      1. Public
      2. PNFP
      3. PFP
      4. Traditional healer
      5. Drug vendor
   3. Misdiagnosis
   4. Missed diagnosis
   5. Health condition at the time of diagnosis
      1. Ambulatory
      2. Emergency admission
   6. Referral
      1. Self-referral vs provider referral
      2. Formal vs informal provider referral
      3. High out-of-pocket expenses
      4. Reasons for referral
         1. Stock-out of diagnostic supplies
         2. For treatment
3. **Pre-treatment interval**
   1. Immediate vs prolonged enrolment into care
   2. Health education
   3. Drug stock-out
   4. Delayed enrolment into care
      1. Fear and disbelief
      2. Alternative diagnosis
      3. Type of health provider visited post-diagnosis
         1. Public
         2. PNFP
         3. PFP
         4. Traditional healer
