# Supplementary material for: Understanding the diagnostic delays and pathways for diabetes in eastern Uganda: A qualitative study
Source: PLoS One. 2021 Apr 21;16(4):e0250421. doi: 10.1371/journal.pone.0250421 (PMC8059813; doi:10.1371/journal.pone.0250421)
Supplement: S1 Table — (DOCX) [file pone.0250421.s002.docx]

S1 Table: Details of participating patients and their diagnostic pathways for diabetes

| **Pathway Typology** | **Patient** | **Age (years), Sex** | **Symptoms** | **Health providers visited** |
| --- | --- | --- | --- | --- |
| **A. Short diagnostic pathway** | *P6* | 62, Male | Generalised body weakness, severe numbness of the feet, genital ulcers, fevers and excessive sweating over three weeks | General hospital |
|  | *P12* | 63, Female | Weight loss, blurry vision, excessive sweating, excessive thirst and frequently urinating all occurring for two months | PFP clinic |
|  | *P15* | 49, Female | Generalised body weakness, fever, excessive thirst and frequently urinating, all happening for one month | HC IV,  General hospital |
| **B. Protracted appraisal** | *P1* | 35, Male | Fever, poor appetite, generalised body weakness, excessive thirst, burning sensations of the feet, and joint pains for one year | General hospital |
|  | *P3* | 42, Female | Weight loss, joint pains, itching of the private parts, and a pricking sensation on the skin of the legs, excessive sweating and excessive thirst within one year | HC III,  General hospital |
|  | *P7* | 52, Female | Dizziness, pricking sensation, headache, feeling that the ears were blocked, and urinating frequently, all happening within three years | General hospital |
|  | *P10* | 68, Male | Joint pain and weakness, excessive hunger, excessive thirst, and frequently urinating over eight months | HC III,  General hospital |
|  | *P17* | 54, Male | Gradual skin discolouration and swelling of the body for seven months | General hospital |
| **C. Protracted appraisal and diagnostic intervals** | *P2* | 32, Female | Severe itching of the private parts, burning sensation of the feet, loss of weight, pain in the hands, blurred vision over two years | Drug vendor,  Traditional Birth Attendant,  General hospital |
|  | *P5* | 31, Female | Fever, abdominal distention, excessive thirst, urinating frequently and blurred vision for nine months | PFP clinic,  HC IV,  Traditional healer,  General hospital |
|  | *P8* | 55, Female | Feverish feeling, mild dizziness, excessive thirst and frequent micturition, loss of consciousness all happening within one year | HC IV,  Traditional healer 1,  Traditional healer 2,  General hospital |
|  | *P9* | 67, Female | Headache, generalised body pains, dizziness, itching and numbness of legs for one year | HC III,  HC IV,  General hospital |
|  | *P11* | 46, Male | Burning sensation of the feet, urinating frequently, and loss of weight and headache over one year | Traditional healer 1,  Traditional healer 2,  Traditional healer 3,  Regional referral hospital |
|  | *P16* | 53, Female | Sharp pains in the legs, excessive thirst, urinating frequently, and high body temperatures over one year | PFP clinic 1  PFP clinic 2  PFP clinic 2  HC IV |
| **D. Delayed treatment** | *P4* | 56, Female | fever, generalised body pain, cough, difficulty in breathing, urinating frequently, excessive sweating, loss of consciousness over one month | HC III,  Traditional healer 1,  Traditional healer 2  General hospital |
|  | *P13* | 52, Male | Generalised body weakness, excessive thirst and frequently urinating, all happening over six months | PFP clinic,  PNFP clinic 1,  HC IV  PNFP Hospital |
|  | P14 | 34, Male | Excessive thirst and frequently urinating over six months | PNFP clinic 1,  PFP clinic,  PNFP clinic 2,  PNFP hospital |
